# 

**Published:** 1915-02-27

**Authors:** 


### A VETERAN MEDICAL OFFICER OF HEALTH.

There passed away on Tuesday, February 16, at the age of eighty-seven, one of the oldest medical officers of health in the person of Dr. Charles Harper, who for thirty-two years has held that position to the Bath Rural District Council. Dr. Harper, who was an old London Hospital man, took his M.R.C.S. Eng. in 1849 and his L.R.C.P. Edin. in 1859, and became a surgeon in the Royal Navy in 1853, being appointed five years later to the Royal Naval Hospital at Stonehouse. He saw active service in the Baltic Campaign, and latterly resided at Batheaston, where he acted as churchwarden.

### A RED CROSS SHEEP.

In order to raise funds for the farmers' branch of the Red Cross Society, a sheep which had been given for the purpose was recently offered for auction at the Bath Cattle Market and sold; the buyer, however, immediately put it up again on behalf of the same cause; it was again sold, this purchaser also following the previous example; and this was done in all fifteen times, with the result that the sum of £53 16s. was obtained for the Society. Such auctions of late have become popular.

### AWARDS TO THE MEDICAL SERVICES.

In connection with the list of honours conferred for services in the field, the following promotions and awards are also announced:—

**Brevet-Colonels.**—Lieut.-Colonels A. J. Luther and C. B. Lawson, M.B.

**Brevet Lieut.-Colonels.**—Majors A. W. Hooper, D.S.O., E. W. Bliss, and M. H. G. Fell.

**K.C.M.G.**—Lieut.-Colonels (temporary Colonels) G. H. Makins, C.B., F.R.C.S. (T.F.), and Sir A. A. Bowlby, C.M.G., F.R.C.S. (T.F.).

**C.B.**—Surgeon-General W. G. Macpherson, C.M.G., K.H.P., M.B., Colonel F. W. C. Jones, M.B., Brevet Colonel Sir W. B. Leishman, K.H.P., F.R.S., M.B., F.R.C.P., Colonel (temporary) Sir J. R. Bradford, K.C.M.G., F.R.S., M.D. (T.F.).

**C.M.G.**—Lieut.-Colonels G. H. Barefoot (temporary Colonel), C. H. Burtchaell, M.B., L. T. M. Nash, B. Forde, M.B., and G. S. McLoughlin, D.S.O., M.B., Majors G. T. K. Maurice and E. T. F. Birrell, M.B.

**D.S.O.**—Majors H. R. Bateman, C. R. Evans, C. M. Goodbody, L. W. Harrison, M.B., H. L. W. Norrington, F. A. Stephens, F. A. Symons, M.B., V. J. Crawford, A. C. Fox, G. J. A. Ormsby, M.D., and N. J. C. Rutherford, M.B.; Captains A. G. Wilson, M.B., F.R.C.S., S. J. A. H. Walshe, M.B., and E. T. Potts, M.D.

**Military Cross.**—Captains C. Kelly, M.D., E. C. Phelan, M.B., W. B. Purdon, M.B., T. H. Scott, M.B., temporary Lieutenant J. R. Waddy, Lieutenants W. Tyrrell, M.B., W. McK. H. McCullagh, M.B., temporary Lieutenant W. W. Ingram, M.B., Lieutenants J. FitzG. Gwynne, M.B., F. C. Davidson, M.B., temporary Lieutenant E. W. Carrington, M.B., Lieutenant T. H. Balfour, M.B., Sergeant-Majors 11000 W. H. Storey, 9515 R. J. Fleming, 14461 A. Baker, 14050 A. Bennett, 11509 J. J. Earp, and 10892 H. J. Reeve.

**Hon. Captain.**—Quartermaster and Hon. Lieutenant E. J. Buckley.

The awards in connection with the **Indian Army** include the following to the Medical Services: *Brevet Colonel.*—Lieut.-Colonel A. H. Moorhead, M.B., I.M.S. *Brevet Lieut.-Colonel.*—Major J. M. Sloan, D.S.O., M.B. *Military Cross.*—Third-class Assistant-Surgeon F. B. A. Braganza.

The **Distinguished Conduct Medal** has also been awarded to the undermentioned warrant officers, non-commissioned officers, and men belonging to the Royal Army Medical Corps, or attached to the field ambulances: 8797 Private G. Fenner, 3rd K.R.R.C. (attached 19th British Field Ambulance), 8313 Private J. W. Folkes, R.A.M.C., 641 Private G. Hill, R.A.M.C. (S.R.), 17907 Private J. E. Hobson, R.A.M.C., 17711 Corporal D. C. Holland, R.A.M.C., 9190 Private W. W. Kirk, R.A.M.C., 4097 Private W. Mosdell, R.A.M.C., 8575 Private C. A. Olds, R.A.M.C., 19306 Private C. Speller, R.A.M.C., 8335 Private A. Spencer, R.A.M.C., 11029 Quartermaster Sergeant A. Spowage, R.A.M.C, 7906 Private M. Sullivan, R.A.M.C., and 8310 Private J. Webster, R.A.M.C.

### Gifts and Bequests.

The Treasurer of the Bradford Royal Infirmary has received a cheque for £250 in payment of a legacy bequeathed by the late Mr. Jonathan Aykroyd, of 1 Carlton Villas, Heaton, Bradford.

Mrs. Nathan Atkinson, of Southport, formerly of Bradford, who died on February 1, 1915, has, under her will, bequeathed the residue of her estate to be divided equally between the Royal Infirmary, the Royal Eye and Ear Hospital, St. Catherine's Home, and the Royal Institution for the Blind, all of Bradford. It is anticipated that the share of each of the four institutions named above will amount to £1,500.

HOSPITAL CHAIRMAN'S BEQUESTS.

The late Mr. Isaac Samuel, J.P., once prominent in the public and commercial life of Cardiff, and the chairman of the Finance Committee of King Edward VII.'s Hospital, has bequeathed 100 guineas to that institution. His other gifts to charities include :—£50 to the Cardiff Institute for the Blind; £50 to the Cardiff Institution for Deaf and Dumb; £50 to the Queen Victoria Jubilee Nurses' Institution (Cardiff branch).

### The Book Market.

LIST OF NEW BOOKS RECEIVED FROM THE FOLLOWING PUBLISHERS:—

A. and C. Black: "Medical Applied Anatomy for Students and Practitioners." By T. B. Johnston, M.B. 7s. 6d. net.

J. and A. Churchill: "Squire's Pocket Companion to the British Pharmacopœia." Second edition. 10s. 6d. net.

W. Heinemann: "Student's Text-book of Hygiene." By W. J. Wilson, M.D., D.Sc. 8s. 6d. net.

H. K. Lewis: "The Extra Pharmacopœia." Sixteenth edition. By W. H. Martindale, Ph.D., Ph.C., F.C.S., and W. Wynn Westcott, M.B., D.P.H. Vol I., 14s. net; Vol. II., 7s. net.

J. B. Lippincott Co.: "Mental Medicine and Nursing." By R. H. Chase, A.M., M.D. 6s. net.

Smith, Elder and Co.: "An Index of Symptoms, with Diagnostic Methods." Fifth edition. By R. W. Leftwich, M.D. 10s. 6d. net.

**Institutional and Public Reports.**

The Australian Institute of Tropical Medicine, Townsville, Queensland: First Half-yearly Report for 1914.

Whitehaven and West Cumberland Infirmary: Report for the Year 1914.

Essex County Hospital, Colchester: Report for 1914.

EMERITUS LECTURE IN MEDICINE.

Sir Richard Douglas Powell, Bart., M.D., will deliver an Emeritus Lecture, entitled "A Case of Congenital Heart Disease," on Friday, March 5, at 3 P.M., in the New Theatre of the Medical School, Middlesex Hospital.

SECRETARIAL CHANGES AT BRADFORD.

The vacancy on the secretarial staff of the Royal Infirmary, Bradford, caused by the appointment of Mr. Alfred Sugden as secretary to the Royal Salop Infirmary, Shrewsbury, has been filled by the promotion of Mr. Henry Maw to the post of cashier and assistant secretary. Mr. J. Bates has been appointed canvasser and collector of subscriptions.

RATES OF SUBSCRIPTION: Twelve months, 6/6; Six months, 4/-; Three months, 2/-, post free to any address in the United Kingdom. Abroad, Twelve months, 8/8; Six months, 5/-; Three months, 2/6, post free.—Address The Subscription Dept., "THE HOSPITAL," The Hospital Building, 28/29 Southampton Street, Strand, London, W.C.

**28/29 SOUTHAMPTON STREET, STRAND, LONDON, W.C.**

